# Explicit and Implicit Devaluation Effects of Food-Specific Response Inhibition Training

**DOI:** 10.5334/joc.256

**Published:** 2023-01-17

**Authors:** Loukia Tzavella, Christopher D. Chambers

**Affiliations:** 1 Cardiff University Brain Research Imaging Centre, CF24 4HQ, UK

**Keywords:** response inhibition, training, go/no-go, devaluation, priming, food

## Abstract

The overvaluation of reward-associated stimuli such as energy-dense foods can drive compulsive eating behaviours, including overeating. Previous research has shown that training individuals to inhibit their responses towards appetitive stimuli can lead to their devaluation, providing a potential avenue for behaviour change. Over two preregistered experiments, we investigated whether training participants to inhibit their responses to specific foods would be effective in reducing their evaluations when these were assessed using both explicit and implicit measures. Participants completed an online session of go/no-go training with energy-dense foods that were consistently associated with either responding (go) or inhibiting a response (no-go). An ‘explicit’ devaluation effect was expected as a reduction in self-reported liking from pre-to post-training for no-go items compared to both go items and foods that were not presented during training (untrained items). An ‘implicit’ devaluation effect was then measured using the affective priming paradigm, by comparing differences in reaction times for congruent and incongruent trials (i.e., priming effects) between food primes. Experiment 1 revealed conclusive evidence for small-to-medium devaluation effects both in terms of explicit ratings and priming effects. We also observed that the priming effect for no-go items was close to zero. Experiment 2 successfully replicated most of the preregistered and exploratory outcomes from Experiment 1 except for the priming effect for untrained items. Potential explanations for this discrepancy are discussed but overall, these findings provide further support for a devaluation effect of response inhibition training. To our knowledge, our study provides the first evidence that training-induced devaluation can potentially be captured by affective priming measures, but more research is needed to further assess their sensitivity before they can be used to elucidate the mechanisms of action underlying devaluation effects.

The overvaluation of reward-associated stimuli in our environment can be associated with certain behaviours that are incompatible with long-term goals, such as excessive smoking, drinking, overeating and gambling (e.g. Clark et al., 2013; Davis et al., 2007; Grant et al., 2015; Hogarth et al., 2010; Rothemund et al., 2007; Stice et al., 2013; Versace et al., 2011). One promising development for reducing the value of target stimuli is cue-specific inhibitory control training (Jones et al., 2018; Stice et al., 2017; Turton et al., 2016). A convergence of evidence indicates that when individuals are trained to inhibit or withhold motor responses towards reward-related cues, a *devaluation effect* can be observed for trained stimuli (e.g. alcohol cues; Houben et al., 2011, 2012; food stimuli; Veling et al., 2013a; Chen et al., 2016; smoking-related cues; Scholten et al., 2019; geometric shapes; Wessel et al., 2015, 2014). Devaluation effects in applied research are often assessed via explicit self-report measures (e.g., Chen et al., 2016; Chen, Veling, Dijksterhuis, et al., 2018; Lawrence et al., 2015; Veling et al., 2013a) which may increase participants’ awareness of the study aims (also see Wessel et al., 2014) and/or introduce strategic responding and response bias (see Podsakoff et al., 2003). Although implicit measures can potentially address these concerns, studies using such measures have yielded mixed findings in food and alcohol research (see Jones et al., 2016). In this study we investigated whether devaluation effects would be observed when both explicit and implicit measures of liking (affective priming paradigm; Fazio et al., 1986; Fazio & Olson, 2003; Hermans et al., 2001; Klauer & Musch, 2003) were employed after food-specific response inhibition training.

## Devaluation effects and response inhibition training

Inhibitory control training studies in the food and alcohol domain most commonly implement tasks adapted from the go/no-go (GNG; Donders, 1969; Newman & Kosson, 1986) and stop-signal paradigms (Lappin & Eriksen, 1966; Logan et al., 1984), which both involve participants inhibiting a motor response towards a specific stimulus/cue. In the stop-signal task, a signal is presented after a dynamically-adjusted delay on a minority of trials, whereas during GNG training, no-go cues generally appear on half of the trials and the cue onset is not adjusted to maintain task difficulty. These differences in task parameters suggest that GNG training does not necessarily draw on top-down inhibitory control (Veling et al., 2017; see also Wessel et al., 2014; Verbruggen & Logan, 2008) and participants may not need to inhibit a response after action initiation but rather refrain from initiating a response when a cue is perceived (action cancellation vs action restraint; Eagle et al., 2008). Regardless, studies have shown that GNG training is associated with larger effects on behavioural outcomes (Allom et al., 2016; Jones et al., 2016).

Studies that tailor these paradigms to eating-related cues have shown promising findings as training has been associated with reduced food intake (Adams et al., 2017; Houben & Jansen, 2011, 2015; Lawrence et al., 2015), altered food choices (Chen et al., 2019; Porter et al., 2018; Veling et al., 2013a, 2013b) and increased weight loss (Lawrence et al., 2015; Veling et al., 2014). The specific mechanisms of action behind these outcomes have not yet been elucidated but a prominent explanation for training effects is *stimulus devaluation* (Veling et al., 2008, 2017; Stice et al., 2016; see also ‘stimulus-stop associations’; Verbruggen et al., 2014; Best et al., 2016). The definition and operationalisation of value can vary in this context (e.g., willingness to pay; Schonberg et al., 2014; attractiveness and tastiness; Veling et al., 2013a; Lawrence et al., 2015) but in this study we focus on evaluations of foods that relate to overall liking (e.g. image attractiveness, tastiness etc). Studies that train individuals to withhold their responses to energy-dense foods have provided support for a robust devaluation effect, which is typically defined as the negative difference between changes in subjective evaluations from pre-to post-training for stimuli consistently associated with response inhibition (*no-go*) between groups and/or relative to stimuli that are presented on go trials (*go*) as well as stimuli that are not included in the training task (untrained; Chen et al., 2016; Chen, Veling, Dijksterhuis, et al., 2018; Lawrence et al., 2015; Veling et al., 2013a).

## Implicit measures & methodological considerations

Although there is evidence to suggest a robust and replicable devaluation effect of response inhibition training for a range of stimuli (e.g., Chen et al., 2016; Houben et al., 2012; Houben & Giesen, 2018; Houben & Jansen, 2011; Johannes et al., 2021; Scholten et al., 2019; Wessel et al., 2014), a recent meta-analysis showed that when studies use the variants of the implicit association test (IAT; Greenwald et al. 1998) to capture affective evaluations, this effect is no longer reliably observed (Jones et al., 2016). For example, Adams et al. (2017) employed single-category IATs with chocolate images (trained vs untrained items) and did not find evidence for an effect of training on implicit food attitudes relative to double-response training (Study 1). Participants in this task are asked to categorise words as quickly and as accurately possible according to different pairings of labels and attribute categories (e.g. ‘chocolate + pleasant’ vs ‘neutral’) and their responses are assumed to reflect automatic affective evaluations as time constraints may prevent conscious or controlled processing (Hermans et al., 2001). However, the explicit categorisation of stimuli may alert participants to the aim of the task allowing for strategic responding to occur and/or result in responses that reflect the evaluation of category labels rather than the stimuli of interest (De Houwer, 2001; Fazio & Olson, 2003).

In this study we employed the affective priming paradigm (APP) as an alternative to the IAT primarily because it requires participants to respond to the valence of words (targets) that are *not* semantically relevant to specific stimuli (primes) that are presented for a very short duration (e.g. <300 ms) and instructions encourage participants to ignore the stimuli and their content altogether (also see Klauer & Musch, 2003; Wentura & Degner, 2010). Evidence for the utility of the APP as an implicit, or indirect, measure of food evaluations has been provided by previous studies (e.g., Lamote et al., 2004; Roefs, Herman, et al., 2005; Roefs, Stapert, et al., 2005). Although it is unclear whether this paradigm can capture differences in the strength of the evaluations (Lamote et al., 2004; see also Herring et al., 2013), priming effects have been demonstrated for acquired attitudes in a laboratory-based evaluative conditioning procedure aimed to increase sensory liking for selected foods (Verhulst et al., 2006). For the present study we adopted the evaluative categorisation task variant of the APP which has been found to reliably measure food liking both in laboratory and online cohorts (Tzavella et al., 2020).

## The present study

The use of implicit measures in applied research either as primary or secondary outcomes may complement existing evidence for training-induced devaluation effects, as these are commonly assessed using explicit self-report methods which can be associated with response bias and demand characteristics (Podsakoff et al., 2003; see also Wessel et al., 2014). Importantly, employing measures that may be sensitive to pre-conscious or automatic affective evaluations such as the APP could shed light into the current theoretical accounts of devaluation effects (Behaviour Stimulus Interaction theory; Veling et al., 2008; hard-wired connection between go and stop systems with Pavlovian appetitive and aversive centres; Verbruggen et al., 2014; see also review in Veling et al., 2017).

In Experiment 1 we tested several preregistered hypotheses regarding the effects of GNG training on explicit and implicit food evaluations. We expected stimulus devaluation to occur for foods associated with response inhibition during training. The devaluation effect was defined as a negative change in liking from pre-to post- training for two contrasts with different baselines (no-go items vs go and untrained items; c.f. Chen et al., 2016). A novel research question was whether devaluation effects could be observed in terms of food priming effects when the APP was employed as an implicit measure of liking. Implicit food evaluations were therefore defined based on reaction time (RT) priming effects and compared across training conditions (go, no-go, untrained). In Experiment 2 we aimed to replicate the findings from both preregistered and *post hoc* exploratory analyses of the original experiment.

## Experiment 1

### Hypotheses

All hypotheses, as outlined below, and respective statistical tests were preregistered prior to data collection (Tzavella & Chambers, 2019; https://osf.io/c6z53). There were no deviations from the study protocol.^1^

H1. Training would have an effect on *explicit* food evaluations:

H1a. The change in liking ratings from pre-to post-training would be negative for *no-go* foods and greater in magnitude compared to the change in liking ratings for *go* foods.

H1b. The change in liking ratings from pre-to post-training would be negative for *no-go* foods and greater in magnitude compared to the change in liking ratings for *untrained* foods.

H2. Training would have an effect on *implicit* food evaluations:

H2a. The RT priming effect for *no-go* foods would be reduced compared to the RT priming effect for *go* foods.

H2b. The RT priming effect for *no-go* foods would be reduced compared to the RT priming effect for *untrained* foods.

H3. Correct RTs on congruent trials would be on average lower compared to correct RTs on incongruent non-food prime trials.

### Method

#### Participants

A total of 140 participants recruited via Prolific (https://www.prolific.co/) and personal communication were assessed for eligibility in the study (see **S1**). Participants had to be at least 18 years old or older, speak English as their first or second language, have normal or corrected-to-normal vision and report no past and/or current history of eating disorders. Participants were not eligible to participate if they were dieting to lose weight and/or taking diet pills at the aim of the study. Participants also had to report no major hearing impairments that would prevent them from hearing the tones presented in the study. Pre-screening on Prolific specified that only individuals currently residing in the UK could participate, as the branded foods included in the study might not have been popular in other countries. A total of 120 eligible participants completed the study and received either monetary compensation (Prolific) or a prize draw entry for a £15 online shopping voucher. The study was approved by the local Research Ethics Committee at the School of Psychology, Cardiff University.

#### Sampling plan

In this experiment we employed an open-ended Sequential Bayes Factor (SBF) design (Schönbrodt et al., 2017) with a maximum sample size (*nmax*) of 130 and a minimum sample size (*nmin*) of 50. Data collection would continue until either the selected evidential threshold for preregistered hypotheses (H1, H2, H3) had been reached, or *nmax* had been met. For convenience in the interpretation of evidence we followed previous guidelines and considered a threshold of *BF*_10_ ≥ 10 would indicate strong evidence for the alternative hypothesis compared to the null and vice versa for *BF*_01_ ≥ 10. To assess the probability of this SBF design generating misleading or inconclusive evidence we performed a Bayes Factor Design Analysis (BFDA; Schönbrodt & Stefan, 2019; Schönbrodt & Wagenmakers, 2018). Further details about the SBF stopping rule and BFDA are presented in the Supplementary Information (SI; see **S2**).

#### Procedure

**Figure 1:**
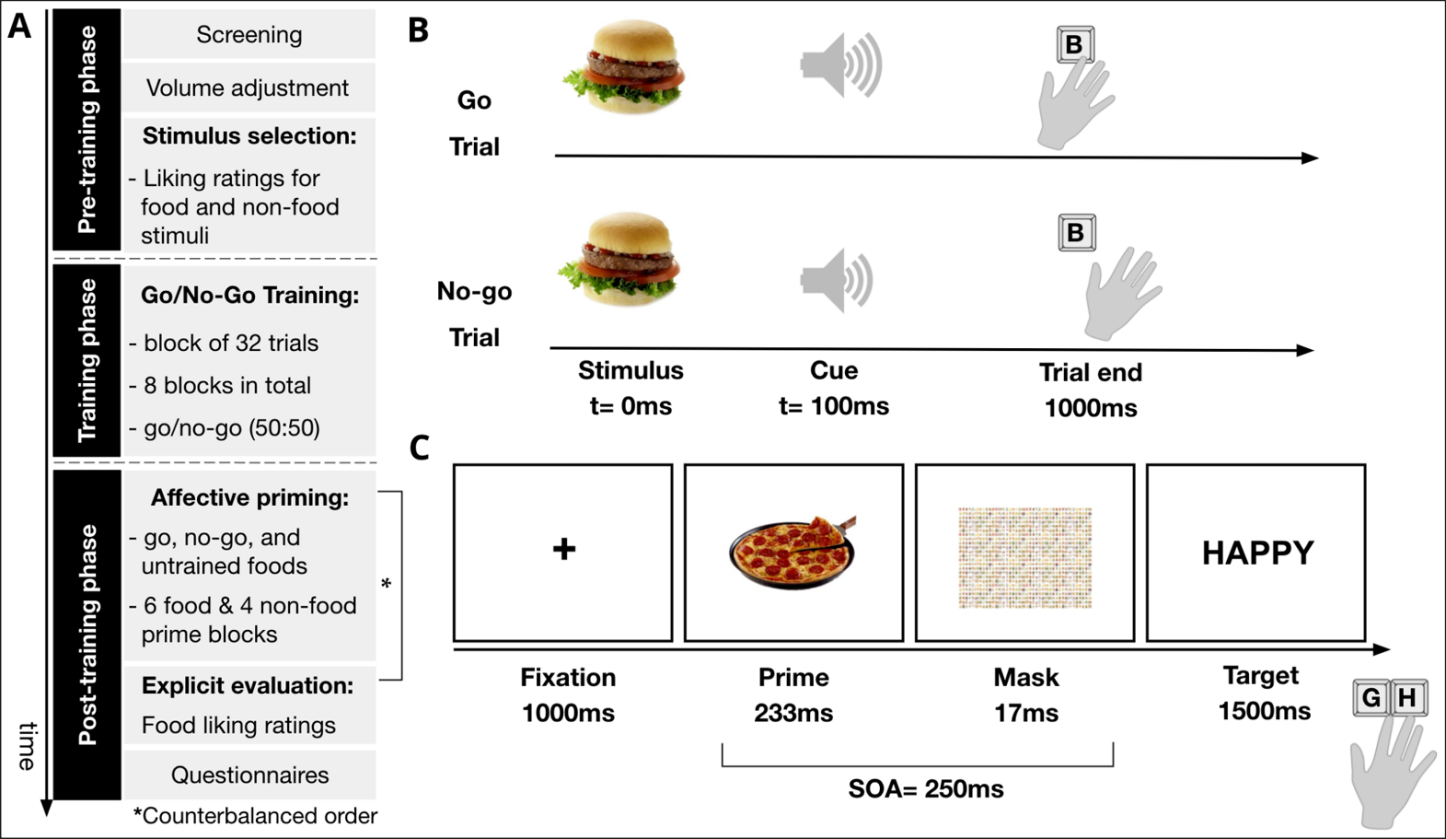
Schematic of study procedure, go/no-go training and affective priming paradigm **A.** After screening, eligible participants adjusted the volume at which they would hear the tones (cues) during training. In the pre-training phase, they provided liking ratings for food and non-food stimuli and stimuli were selected for training and the affective priming paradigm (go, no-go, and untrained foods). Participants performed eight blocks of the go/no-go training task in total and in each block of 32 trials, go and no-go foods appeared with equal probability (50:50). In the post-training phase, the affective priming paradigm (APP) and food ratings were presented in counterbalanced order across participants. The APP consisted of both food and non-food prime blocks, presented in a fixed order and food prime blocks included go, no-go and untrained foods. For the explicit evaluation of foods, participants provided liking ratings and at the end of the study several questionnaires were completed. **B.** In the go/no-go training task, participants were asked to press “B” to respond on trials where a specific cue (tone) was presented (i.e., no-signal, or go trial). When another cue was heard, participants had to refrain from responding (i.e., signal, or no-go trial). The cues, which were randomly assigned to trial types across participants, were presented 100 ms after stimulus onset and lasted 300 ms. The trial duration was fixed to 1000 ms. **C.** In the APP, participants responded to positive and negative targets by pressing the “G” and “H” keys (counterbalanced) which were preceded by food and non- food primes. The maximum reaction time was 1500 ms and the stimulus-onset asynchrony (SOA) between primes and targets was 250 ms, including the mask duration (17 ms).

The overall study procedure is shown in Figure 1A. Participants were initially screened for eligibility, provided their consent, and proceeded to adjust the volume at which the tones (cues) for the go/no-go training task would be presented. An initial explicit evaluation task was performed to record pre-training ratings and to select the most appetitive foods for the training paradigm (go, no-go foods; see *Pre-training ratings & stimulus selection*) and APP (untrained foods). Participants then rated non-food stimuli and the most liked categories were used in the non-food prime APP blocks (manipulation check). During the training phase of the study, participants performed eight blocks of the GNG training task with a short practice block in the beginning (see *Go/no-go training task*).

After training completion, the APP (see *Affective priming paradigm*) and explicit evaluation task (see *Post-training ratings*) were presented in a counterbalanced order across participants. Food prime (APP_FOOD_) and non-food prime APP blocks (APP_NONFOOD_) were presented in a fixed order, such that a non-food prime block was always followed by two food prime blocks. A short practice block was also provided for the APP. In the explicit evaluation task after training, participants rated all foods (go, no-go, untrained) with a total of 24 trials. At the end of the study, questionnaire measures were completed (see *Questionnaires*) and participants were debriefed on the study aims. The study was run via Inquisit Web and was programmed using Inquisit Lab 5 (Millisecond Software, 2016).

#### Pre-training ratings & stimulus selection

In an initial explicit evaluation task, participants were presented with 50 foods that were high in fat, sugar and/or salt. Stimuli for the study have been obtained from the food-pics database (Blechert, 2019; Blechert et al., 2014) and other sources (see **S3** for details). The food stimuli appeared in random order and participants rated them according to how much they liked them at the time (“How much do you like this food right now?”) on a visual analogue scale (VAS) ranging from –100 to 100, always centred at zero. The exact values on the VAS were not visible to the participants. The foods with the highest ratings were ranked from 1 to 24 and were assigned to three sets of eight foods, in a manner that ensures that the average explicit liking values were as matched as possible for all sets (c.f. Chen et al., 2016). These three sets were then randomly selected as go, no-go or untrained foods. Following the explicit evaluation of all foods, participants were presented with 25 positive non-food stimuli (e.g., kittens) and were asked to rate how much they like them (“How much do you like this image?”). The stimuli with the maximum rating were assigned as primes in the non-food prime APP blocks (N = 12). There were two exemplars per food and non-food category in both the training task and APP.

#### Go/no-go training task

The training paradigm employed in this study was a go/no-go task adapted from Chen et al. (2016). In GNG training, participants are either required to respond to (food) stimuli (i.e., go trials) or inhibit their responses (i.e., no-go trials) towards them. In this task, go and no-go trials appeared with equal probability (50% no-go) and each trial began with the central presentation of a food stimulus, which was followed by a cue at 100 ms (see Figure 1B). The auditory cue was either a 440Hz or 1000Hz tone presented for 300 ms and was randomly assigned to either go or no-go trials across participants. To ensure that all participants could hear the tones properly, in the beginning of the study they were asked to adjust the volume at which the tones would be played and the volume, which could be different for each tone, was changed automatically via the programmed script. Responses on each trial were determined by the assigned cue. On go trials, participants needed to press the “B” key using their index finger as fast as possible after cue onset and on no-go trials, they were instructed not to respond at all. The food stimulus remained on the screen for the total trial duration (i.e., 1000 ms) to control for visual exposure time within and across participants. The inter-trial interval (ITI) randomly varied from 800 ms to 1500 ms, in intervals of 100 ms. This was a deviation from the task design adapted by Chen et al. (2016) aimed to slightly reduce the total duration of the GNG task for online data collection.

Each GNG training block consisted of 32 trials and participants performed eight blocks in total (256 trials). As described in the previous section, randomly assigned foods were presented on go and no-go trials, with two exemplars for each food category. Therefore, each food category had 16 repetitions across blocks and specific exemplars were repeated eight times in total. Participants first completed a short practice block (16 trials) with remaining stimuli from the selection process and accuracy feedback appeared after each trial. The screen background was white and food stimuli had relative dimensions according to the participant’s display resolution (40% width × 40% height). A short break was provided after four blocks of training.

#### Affective priming paradigm

The affective priming paradigm (APP) was adapted from a previous study that assessed its utility as an indirect measure of food liking (Tzavella et al., 2020). It involved an evaluative categorisation task, in which participants had to categorise words (i.e., targets) as either positive or negative, as fast, and as accurately as possible, when these were preceded by selected stimuli (i.e., primes). The primes were presented supraliminally with a stimulus-onset-asynchrony of 250 ms, as shown in Figure 1C. In each trial, participants were asked to focus on a central fixation point (1000 ms) and the prime was presented for 233 ms. The prime was followed by a backward mask (17 ms) and the target then stayed on the screen for the maximum reaction time (maxRT) of 1500 ms. The response keys “G” and “H” were randomly assigned to positive and negative targets across participants. Participants had to respond with the index and middle fingers of their preferred or dominant hand. A response was considered correct if participants categorised the target correctly before maxRT was reached. RTs were recorded from prime onset, at 1250 ms, and each trial ended either when a response was registered, or the total trial duration was reached.

Each APP block consisted of 48 trials and participants completed six blocks in total. All food categories for each training condition (go, no-go, untrained; 16 trials each) were included and represented by two exemplars, which were randomly assigned to interleaving blocks to be paired with either positive or negative targets. For example, in block 1 the first exemplar of a go food category was paired with a positive target and the second with a negative target, whereas in block 2 this assignment was reversed. This ensured that both food exemplars were presented as primes in both congruent (positive target) and incongruent trials (negative target). The APP included 24 positive and 24 negative targets (see **S4**) that we considered could be “unambiguously” categorised (Wentura & Degner, 2010).

A manipulation check for the task as an indirect measure of liking was employed in line with previous work (Tzavella et al., 2020). In four blocks of 24 trials, non-food stimuli that had the highest liking rating were assigned as primes and were paired with either positive (congruent) or negative targets (incongruent). All targets appeared randomly across consecutive blocks. For non-food primes, there were a total of 48 observations per design cell (i.e., affective congruence). The non-food prime blocks (APP_NONFOOD_) were presented in between two food prime APP blocks (APP_FOOD_) and the order was fixed across participants: one APP_NONFOOD_ block, two APP_FOOD_ blocks, …, one APP_NONFOOD_ block.

The screen background for the APP was white, consistent with the GNG task design, and all words were capitalised. Participants completed 16 practice trials and the primes were foods that had not been assigned to an experimental set during stimulus selection. Feedback was provided for both speed and accuracy. After three APP blocks participants received a short break and were reminded of the main task instructions and response key assignments.

#### Post-training ratings

Participants rated all foods from the go, no-go, and untrained conditions after training (random order) and food categories were represented by the same exemplars from the initial explicit evaluation task. Instructions for participants highlighted that some pictures may be the same as in the first rating task, but they should indicate how much they liked them at that specific time. Instructions for both pre- and post-training ratings encouraged participants to pay attention to the specific pictures depicting each food: “For instance, you may generally like a certain type of crisps but not find this particular flavour very appealing at this specific time [..]”.

#### Questionnaires

Several demographic as well as trait and state variables were recorded at the end of the study, including gender, ethnicity, hunger levels (“How hungry are you right now?”; 1 = “Not at all” to 9 = “Very”), hours since last meal (“Less than 1 hour ago”, “1–3 hours ago”, “3–5 hours ago”, “More than 5 hours ago”) and dietary preferences (e.g., vegetarian, vegan; and open-ended option). Body height and weight were self-reported to calculate the participant’s body-mass index (BMI; kg/m^2^).

After answering these questions, participants completed a follow-up study questionnaire which assessed their perceived performance in the APP (e.g., strategic responding), number of interruptions during the study (Waters & Li, 2008) and included attention as well as instruction manipulation checks (Kees et al., 2017). The original questionnaire (see Tzavella et al., 2020) in this study had additional questions that attempted to capture participants’ awareness of study hypotheses/aims prior to completion and stimulus-response contingencies in the training task. The follow-up study questionnaire and additional questionnaires that were added as part of an assignment for BSc Psychology at Cardiff University can be found in the SI (**S5**).

### Analyses

#### Measures & Indices

For APP performance and calculated priming effects, median RTs were obtained from correct trials only, for each participant and each design cell (e.g., congruent go food trials). Medians were preferred instead of means as they are less sensitive to outliers and would indicate central tendency more accurately in the expected positively-skewed RT distributions. Priming effects (ΔRT) were calculated as the change in median RTs from incongruent to congruent trials (medianRT_INC_ – medianRT_CON_) for each training condition (go, no-go, untrained). For explicit evaluations pre- and post-training, mean ratings for each condition were calculated (go, no-go, and untrained foods). Difference scores of these means were finally calculated for each training condition (i.e., ΔLiking_GO_, ΔLiking_NOGO_, ΔLiking_UNTRAINED_). Negative difference scores (post – pre) would reflect a reduction in liking from pre-to post-training. Other descriptive measures of APP and GNG task performance were also recorded for data exclusions and exploratory analyses, such as the inspection of potential speed-accuracy trade-offs in the APP (see **S7**).

#### Data exclusions

Accuracy was inspected for both the GNG and APP tasks. Participants with error rates (ER) greater or equal to 0.4 from within either the set of critical food prime or non-food prime trials were excluded from all respective analyses. For the GNG task, participants who had a proportion of successful inhibitions (i.e., correct no-go responses; PC_NOGO_) lower than 0.65 would be excluded, as it has been shown that this is an important moderator for training effects (see section 3.3 in Jones et al., 2016). Participants who did not complete all the tasks/measures of the study critical to confirmatory hypotheses (GNG, APP, explicit evaluation task) were not included in preregistered analyses. Further data exclusions would be implemented based on the timing accuracy in APP trials (i.e., prime and/or mask duration delayed by two or more refresh rates at 17 ms). Trials with such delays would be discarded from the data, but if participants had more than 25% trials removed, they would be excluded from all analyses. All criteria outlined here were preregistered prior to data collection and exclusions can be found in the SI (see **S1**).

#### Preregistered analyses

Data pre-processing and analyses were conducted in R (R Core Team, 2017) using RStudio (RStudio Team, 2016) and scripts are publicly available at https://osf.io/6bsnv/. All confirmatory hypotheses were tested using directional Bayesian paired-samples t-tests (Rouder et al., 2009), as shown in Table 1. The prior with the √2/2 scale parameter for the half-Cauchy distribution was used for all t-tests. We conducted further checks to assess how robust the results were to the choice of this ‘default’ prior (see **S8**). Shapiro-Wilk tests were performed to check for potential violations of the normality assumption and in case of violation^2^ additional analyses based on log-transformed RTs for H2 and H3 would be reported in a supplementary manner. Together with alternative analyses for normality violations under H1, which were not preregistered, supplementary statistics can be found in the SI (see **S6**).

**Table 1:** Preregistered t-test results for hypotheses in Experiment 1.


					95% CI FOR *D_AV_*	

Preregistered t-test	*BF*10	*T*(112)	*p*	*D_AV_*	LOWER	UPPER

H1a. ΔLiking_NOGO_ < ΔLiking_GO_	109.42	–3.68	<.001	–0.38	–0.59	–0.17

H1b. ΔLiking_NOGO_ < ΔLiking_UNTRAINED_	678.73	–4.22	<.001	–0.39	–0.58	–0.20

H2a. ΔRT_NOGO_ < ΔRT_GO_	44.30	–3.39	<.001	–0.37	–0.58	–0.15

H2b. ΔRT_NOGO_ < ΔRT_UNTRAINED_	30.06	–3.26	0.001	–0.32	–0.51	–0.12

H3. RT_CON_ < RT_INC_ (non-food primes)	158.99	–3.80	<.001	–0.16	–0.25	–0.07


ΔLiking: Difference in mean liking ratings from pre-to post-training (post – pre); ΔRT: Difference in median RTs from congruent and incongruent trials (i.e., reaction time priming effect; incongruent – congruent).

### Results

#### Sample characteristics

The final sample for Experiment 1 consisted of 113 participants. All participant exclusions can be found in the SI (**S1**). Overall, the average liking ratings for the food items in each training condition were matched and the majority of participants found the foods appetitive (go: *M* = 52.6, *SD* = 25.1; untrained: *M* = 52.2, *SD* = 25.1; no-go: *M* = 52.4, *SD* = 25.0). The percentage of successful inhibitions was very high across participants (*M* = 96.7, *SD* = 3.5). Descriptive statistics of sample demographics and trait/state variables were recorded for 112 participants. Although the data were collected online the sample was not diverse in terms of ethnicity or background (87.5% White; 7.1% Asian/Asian British; 1.8% Black/Black British/African/Caribbean; 1.8% Mixed or Multiple ethnic groups; 1.8% Other ethnic groups) and age (years; *M* = 26.9, *SD* = 10.7). The proportion of female participants was 57.1% and the proportion of male participants was 42.9%.

Participants were on average not very hungry at the time of the study (*M* = 4.8, *SD* = 2.6). This is consistent with the self-reported hours since last food intake, which were not indicative of elevated appetite, as 49.1% of participants had a meal 1–3 hours before the study and 23.2% had a meal less than one hour before. Most participants did not follow a specific diet (84.8%), while 6.3% reported a vegetarian diet, 3.6% a vegan diet, 3.6% a pescetarian diet and only 1.8% another diet (e.g., ketogenic). Participants’ average BMI was 24.2 kg/m^2^ (*SD* = 5.1; N = 111). Almost all participants indicated that they had no prior knowledge of the study hypotheses/aims (98.2%). After study completion, 36.6% of participants reported an awareness of stimulus-response contingencies for no-go trials during training and most participants (94.6%) passed the attention check in the follow-up questionnaire.

#### Findings from preregistered analyses

**Figure 2:**
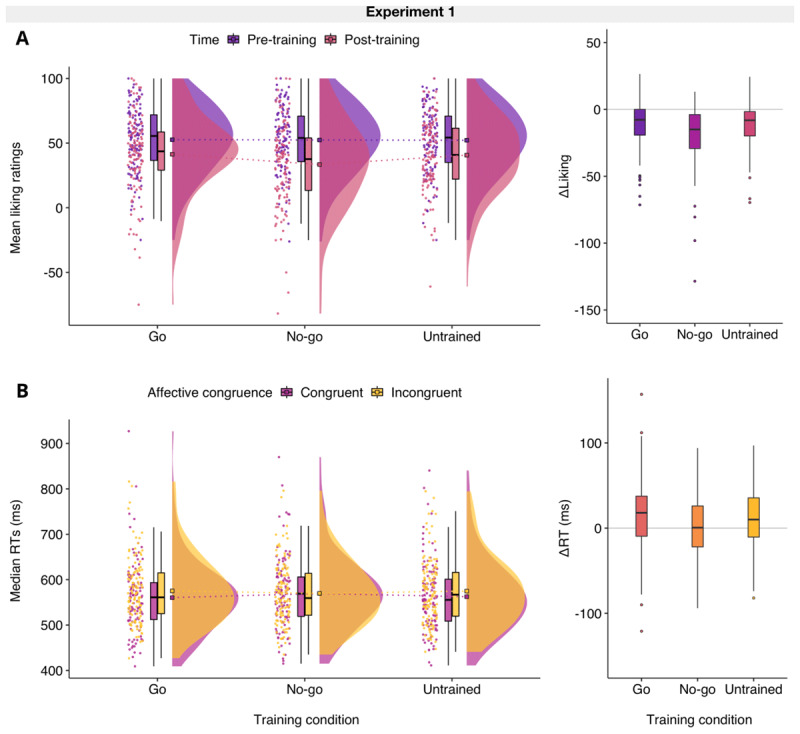
Plots of liking ratings and priming effects in Experiment 1. **A.** The distributions of mean liking ratings indicate that on average all foods were rated less positively after training relative to baseline, but this negative change in explicit evaluations (ΔLiking) was reliably larger for no-go foods compared to both go (H1a) and untrained foods (H1b). **B.** The distributions of individual median reaction times (RTs) from congruent and incongruent trials of the affective priming paradigm (APP) show that positive priming effects were observed for both go and untrained foods. As expected, the priming effect (ΔRT) was lower for no-go foods compared to both go (H2a) and untrained food primes (H2b). Upon closer inspection, the average no-go priming effect was close to zero, even though these foods were rated high on liking before training. *Note*. The ‘split-half violin’ elements in the raincloud plot show smoothed distributions and boxplot vertical lines represent the range, excluding outliers based on the Interquartile Range. Square boxes depict the sample means and the dashed lines show the differences across training conditions.

All results from Bayesian and supplementary frequentist statistical tests can be found in Table 1. There was *extreme* evidence that go/no-go training had an *explicit* devaluation effect on the selected foods when both baselines were examined (i.e., go and untrained foods). Liking ratings pre- and post-training have been visualised using a raincloud plot (Allen et al., 2018, 2019) and together with changes in liking ratings (ΔLiking) across training conditions they can be seen in Figure 2A. As predicted in H1a, the change in liking ratings, from pre-to post- training was negative for no-go foods (ΔLiking_NOGO_; *M* = –18.9, *SD* = 21.9) and greater compared to the change for go foods (ΔLiking_GO_; *M* = –11.3, *SD* = 18.3). Similarly, there was conclusive support for H1b, as the negative change in liking ratings was greater for no-go foods after training relative to untrained foods (ΔLiking_UNTRAINED_; *M* = –11.5, *SD* = 15.6).

The RT priming effects (ΔRTs) for each training condition were also examined and there was *very strong* evidence for an *implicit* devaluation effect (see Figure 2B). H2a was supported and the RT priming effect was lower for no-go foods (ΔRT_NOGO_; *M* = 0.3 ms, *SD* = 38.6) compared to go foods (ΔRT_GO_; *M* = 14.8 ms, *SD* = 40.7). H2b also received conclusive support, as the RT priming effect for no-go foods was lower for untrained foods (ΔRT_UNTRAINED_; *M* = 12.2 ms, *SD* = 37.0) relative to go foods. Finally, the manipulation check for the APP (H3) was successful, as there was *extreme* evidence that participants were on average faster to respond on congruent (*M* = 561.0 ms, *SD* = 77.8) rather than incongruent non-food prime trials (correct RTs only; *M* = 573.1 ms, *SD* = 73.0).

#### Findings from exploratory analyses

Although the difference scores for RT priming effects in each training condition were compared directly in planned paired comparisons, additional exploratory analyses were conducted to examine whether positive effects would be obtained for the two baselines (go and untrained foods). There was *extreme* evidence that participants were on average faster to respond on congruent (*M* = 560.4 ms, *SD* = 77.7) compared to incongruent go food prime trials (*M* = 575.2 ms, *SD* = 72.1) [*BF*_10_ = 195.25; *t*(112) = –3.86, *p* < .001, *d_av_* = –0.20, 95% CI for *d_av_* = –0.30, –0.09]. There was also *very strong* evidence for a positive RT priming effect for congruent (*M* = 562.5 ms, *SD* = 76.7) and incongruent untrained food prime trials (*M* = 574.7 ms, *SD* = 73.4) [*BF*_10_ = 64.58; *t*(112) = –3.52, *p* < .001, *d_av_* = –0.16, 95% CI for *d_av_* = –0.26, –0.07]. However, there was *moderate* evidence for the absence of a positive RT priming effect when no-go foods were examined. Participants were not faster to respond on congruent (*M* = 569.5 ms, *SD* = 78.2) compared to incongruent no-go food prime trials (*M* = 569.8 ms, *SD* = 69.4) [*BF*_01_ = 8.99; *t*(112) = –0.08, *p* = 0.468, *d_av_* < 0.001, 95% CI for *d_av_* = –0.10, 0.09]. The absence of an overall RT priming effect for no-go foods is also illustrated in Figure 2B (boxplot). Supplementary analyses showed no evidence for speed-accuracy trade-offs in the APP (see **S7**), indicating that the RT effects reported here could not be attributed to strategic responding.

### Discussion

Preregistered analyses showed that the change in liking ratings from pre-to post- training was negative for no-go foods and greater in magnitude compared to the change in liking ratings for both go foods and untrained foods. These results suggest that the go/no-go training task employed in this study had an explicit no-go devaluation effect and indirectly replicate the robust devaluation effects observed in other preregistered studies (Chen et al., 2016; Chen, Veling, Dijksterhuis, et al., 2018). Specifically, as the GNG task design was adapted by Chen et al. (2016), it is worth noting that the effect size for a reduction in explicit evaluations for no-go foods compared to go foods (see Table 1) was small-to-medium consistent with the results reported by the authors in Experiment 1.^3^ Chen et al. also reported a medium effect for change in evaluations for no-go foods relative to untrained foods, but in this study, there was only a small-to-medium effect for this comparison as well.

To our knowledge, this is the first study to show that the affective priming paradigm can be successfully applied as an outcome measure for stimulus devaluation after go/no-go training. It was hypothesised that the expected positive RT priming effects for foods would differ between training conditions, so that the priming effect for no-go food primes would be reduced compared to the observed effects for both go and untrained food primes. Although there is not enough evidence to infer whether the magnitude of the APP priming effects can be influenced by the strength of the primes, such as how much participants like the food items (Herring et al., 2013; Lamote et al., 2004), there was very strong evidence for the expected differences between RT priming effects for no-go compared to go and untrained foods. Exploratory analyses indicated that the no-go RT priming effect was close to zero, which may suggest that foods associated with response inhibition during training are evaluated less positively at a pre-conscious level, leading to reduced response facilitation/interference during the APP trials.

## Experiment 2

The primary aim of Experiment 2 was to directly replicate the observed devaluation effects for foods associated with response inhibition, both in terms of explicit evaluations and RT priming effects. All methods for the training task and outcome measures were identical to the original experiment with only a minor change to the number of food items participants could rate in the beginning of the study for stimulus selection. Certain questionnaires which were presented at the end of the study were removed and recruitment was expanded to include participants from Cardiff University. In addition to the main hypotheses in Experiment 1, we also tested hypotheses that were formulated from our *post hoc* analyses (see *Findings from exploratory analyses*).

### Hypotheses

All hypotheses that were introduced for Experiment 2 are listed below and the preregistered study protocol for the replication and extension of findings from Experiment 1 is available at https://osf.io/p6yk9. Note that hypotheses H1, H2, and H3 were identical to Experiment 1.

H4. RT priming effects across training conditions

H4a. Participants will be on average faster to respond on congruent compared to incongruent go food prime trials.

H4b. Participants will be on average faster to respond on congruent compared to incongruent untrained food prime trials.

H4c. Participants will *not* be on average faster to respond on congruent compared to incongruent no-go food prime trials.^4^

### Method

#### Participants & sampling plan

A total of 290 participants who were recruited via Prolific and the Experimental Management System at Cardiff University^5^ were assessed for eligibility. After exclusions, 218 individuals participated in the study (for details see **S1**). All data collection procedures were consistent with those reported for Experiment 1. The sampling plan for Experiment 2 was updated according to a new analysis prior for planned Bayesian t-tests (see *Analyses*). For the replication and extension of findings in this experiment the *nmax* was increased to 200 (after exclusions), but data collection had to be terminated at N = 190 due to time constraints. Details for the BFDA run for Experiment 2 can be found in the SI (see **S2**).

#### Procedure & stimulus selection

The overall study procedure in Experiment 2 was the same, but the questionnaires which were added in Experiment 1 as part of an assignment for undergraduate students were no longer included (see **S5**). The study was run using the latest version of Inquisit Web at the time of data collection and therefore all scripts were updated in Inquisit 6 (Millisecond Software, 2020). The only deviation from Experiment 1 in this replication concerned the number of stimuli participants rated in the pre-training phase. To reduce the total estimated study completion time and ensure that only appealing stimuli were included in the study, the ratings from all participants in Experiment 1 were inspected (N = 120; without data exclusions) and the least liked stimuli were removed. The number of food stimuli was therefore decreased from 50 to 40 and the number of non-food stimuli was 20 rather than 25. Details regarding the specific items and the ratings can be found in the preregistration for Experiment 2 (https://osf.io/p6yk9). This modification does not affect any of the design parameters in the GNG task and APP, which remained the same as in Experiment 1.

### Analyses

All details regarding measures, indices and data exclusions are the same as in the original preregistered experiment but for all analyses the Bayesian t-tests were conducted with updated prior parameters. Based on the effect sizes reported in Experiment 1 and previous recommendations for small-to-medium effects in related research areas (Quintana & Williams, 2018; see also Gronau et al., 2018; Stefan et al., 2019), we adopted a prior with a t-distribution with the location parameter μ set to –0.35 (r = 0.102, df = 3). Bayesian analyses were performed using JASP to allow for non-default prior specification (JASP Team, 2020).

### Results

#### Sample characteristics

The final sample for Experiment 2 consisted of 190 participants (see **S1** for data exclusions). The baseline liking ratings for selected foods in each training condition were matched, although the scores were slightly lower than those in Experiment 1 (go: *M* = 45.9, *SD* = 29.2; no-go: *M* = 46.0, *SD* = 28.6; untrained: *M* = 45.9, *SD* = 28.8). Participants’ accuracy (%) in the GNG was high (*M* = 95.3, *SD* = 5.1) consistent with performance in Experiment 1. Demographics showed that the samples in both experiments were approximately matched on ethnicity or background (N = 187; 80% White; 9.5% Asian/Asian British; 5.3% Mixed or Multiple ethnic groups; 2.1% Black/Black British/African/Caribbean; 1.6% Other ethnic groups; 1.6% did not wish to answer) and age (*M* = 23.6, *SD* = 9.0). In this sample, the proportion of female participants was higher (76.8%), with 21.6% of participants identifying as male and 1.6% as gender-variant/non-conforming. A key difference between the samples in Experiments 1 and 2 would be that most participants for this replication were recruited from the student population at Cardiff University (67.4%).

Participants’ self-reported hunger at the time of the study was not high (*M* = 4.9, *SD* = 2.6), which was expected as 26.8% had a meal one hour before and 44.2% had a meal one to three hours before participation. Most participants were not following a specific diet (80.5%), while 9% were vegetarian, 4.74% were vegan, 3.7% were pescetarian and only 2.1% reported other diets. Participants’ average BMI was 23.8 kg/m^2^ (*SD* = 4.5; N = 189). Most participants (95.2%) did not report any prior knowledge of the study aims/hypotheses. Only 30.7% of participants learned stimulus-response contingencies for no-go trials during training and 94.7% answered correctly on the attention check question.

#### Findings from preregistered analyses

Several hypotheses from Experiment 1 received conclusive support in this replication (H1a, H2a, H3), but results were not consistent for the second baseline in our devaluation contrasts; that is, for untrained foods (H1b, H2b). All results from preregistered t-tests can be seen in Table 2. There was *extreme* evidence that the negative change in liking for no-go foods (*M* = –17.94, *SD* = 22.47) was greater than the change for go foods (*M* = –11.57, *SD* = 19.50), but only *moderate* evidence for a slight difference in magnitude between the change for no-go foods and untrained foods (*M* = –14.97, *SD* = 22.41). There was *extreme* evidence that the RT priming effect for no-go foods (*M* = –0.82, *SD* = 38.57) was lower compared to the priming effect for go foods (*M* = 14.55, *SD* = 40.36), but only inconclusive evidence for the absence of the expected difference in RT priming effects for no-go and untrained foods (*M* = 4.50, SD = 36.80).

**Table 2:** Preregistered t-test results for hypotheses in Experiment 2.


					95% CI FOR *D_AV_*	

Preregistered t-test	*BF*10	*T*(189)	*P*	*D_AV_*	LOWER	UPPER

H1a. ΔLiking_NOGO_ < ΔLiking_GO_	18809.23	–4.70	<.001	–0.30	–0.43	–0.17

H1b. ΔLiking_NOGO_ < ΔLiking_UNTRAINED_	3.35	–2.37	0.010	–0.13	–0.24	–0.02

H2a. ΔRT_NOGO_ < ΔRT_GO_	70522.41	–5.00	<.001	–0.39	–0.55	–0.23

H2b. ΔRT_NOGO_ < ΔRT_UNTRAINED_	0.59	–1.73	0.043	–0.14	–0.3	0.02

H3. RT_CON_ < RT_INC_ (non-food primes)	96875.68	–5.08	<.001	–0.16	–0.23	–0.1

H4a. RT_CON-GO_ < RT_INC-GO_	60993.53	–4.97	<.001	–0.18	–0.25	–0.11

H4b. RT_CON-UNTRAINED_ < RT_INC-UNTRAINED_	0.53	–1.69	0.047	–0.06	–0.13	0.01

H4c. RT_CON-NOGO_ < RT_INC-NOGO_	0.02	0.29	0.615	0.01	–0.06	0.08


ΔLiking: Difference in mean liking ratings from pre-to post-training (post – pre); ΔRT: Difference in median RTs from congruent and incongruent trials (i.e., reaction time priming effect; incongruent – congruent).

Consistent with findings from exploratory analyses in Experiment 1, the preregistered hypotheses H4a and H4c were supported. There was *extreme* evidence that participants were on average faster to respond on go food prime trials when these were congruent (*M* = 572.9 ms, *SD* = 84.1) rather than incongruent (*M* = 587.4 ms, *SD* = 79.7). As expected, the RT priming effect for no-go foods was not observed,^6^ as there was *very strong* evidence that participants were not faster to respond on congruent (*M* = 583.0 ms, *SD* = 81.6) than incongruent trials (*M* = 582.1 ms, *SD* = 79.5). Contrary to findings from Experiment 1 but consistent with the results regarding explicit devaluation, there was inconclusive evidence for the absence of an RT priming effect for untrained foods although descriptively participants were on average slightly faster to respond on congruent (*M* = 579.2 ms, *SD* = 77.6) relative to incongruent trials (*M* = 583.7 ms, *SD* = 77.7).

### Discussion

The aim of Experiment 2 was to directly replicate all findings from Experiment 1 in a confirmatory manner. First, devaluation effects were observed for both the explicit and implicit measures of liking when no-go items were compared to go items and the effects were in the small-to-medium range consistent with Experiment 1. However, the devaluation effects when defined as the negative difference between no-go items and untrained items were smaller for explicit ratings and inconclusive for RT priming effects in Experiment 2.

**Figure 3:**
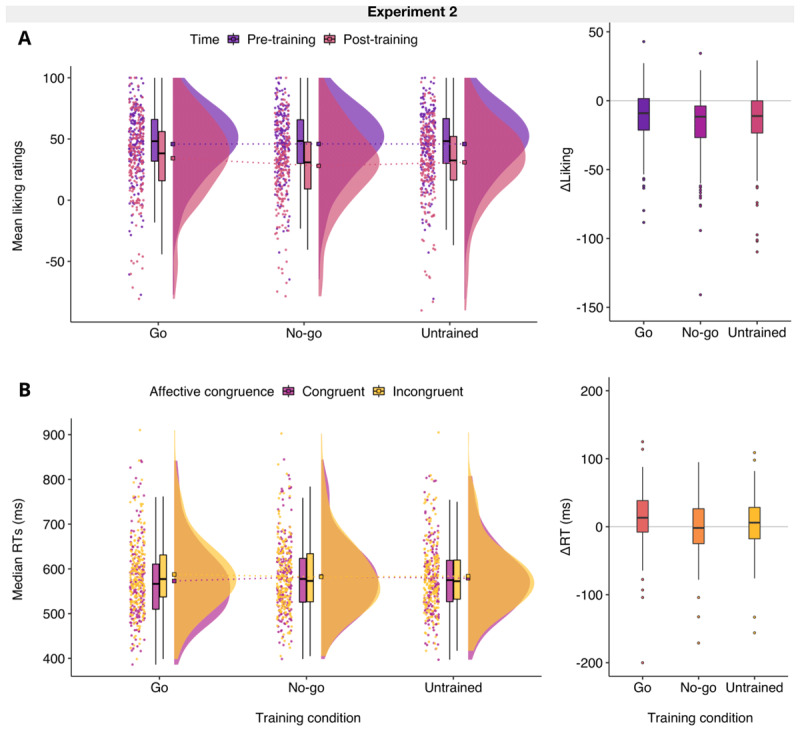
Plots of liking ratings and priming effects in Experiment 2. **A.** The plots show that compared to go foods, the change in liking for no-go (ΔLiking) foods was greater (H1a), but not relative to untrained foods (H1b; see Figure 1A for comparison with Experiment 1). **B.** As expected, participants’ median reaction times (RTs) from congruent and incongruent trials in the affective priming paradigm indicate that a positive priming effect was observed for go foods (H4a), but not for no-go foods (H4c) which have an overall RT priming effect (ΔRT) close to zero, as shown in the boxplot. However, for untrained foods the priming effects in the sample are not as positive as those observed in Experiment 1 (H4b; see Figure 1B). *Note*. The ‘split-half violin’ elements in the raincloud plot show smoothed distributions and boxplot vertical lines represent the range, excluding outliers based on the Interquartile Range. Square boxes depict the sample means and the dashed lines show the differences across training conditions.

Consistent with Experiment 1, the manipulation check for the APP was successful and a positive RT priming effect as observed for go food primes. Importantly, the close-to-zero RT priming effect for no-go food primes was replicated in this experiment, which has implications for the theoretical explanations of training-induced devaluation effects. Specifically, these findings suggest that during training, negative affect is attached to stimuli that are consistently associated with response inhibition (see also Guitart-Masip et al., 2012; Veling et al., 2008; Verbruggen et al., 2014), which could reduce or eliminate response facilitation on congruent trials in the APP (also see RT distributions in Figures 2B and 3B).

## General Discussion

The present study employed a training task adapted from the go/no-go (GNG) training paradigm which has previously been associated with robust devaluation effects of appetitive stimuli (e.g., Houben et al., 2011, 2012; Veling et al., 2013a; Chen et al., 2016; Scholten et al., 2019) to investigate whether such effects would be observed when stimulus evaluations are measured using explicit and implicit measures. In GNG training, various appetitive foods that were matched on their overall liking ratings at baseline were assigned to either no-go trials or go trials. We expected that the change in explicit evaluations for items consistently associated with response inhibition (no-go) would be negative, and greater in magnitude compared to the change for items that were paired with responding (go) and items that were never presented during training (untrained). In the affective priming paradigm participants’ reaction times on congruent (positive target) and incongruent (negative target) trials were compared for no-go food primes relative to go and untrained food primes.

### Did food-specific response inhibition training lead to an ***explicit*** devaluation effect?

Yes. Both in Experiment 1 and Experiment 2 there was evidence for training-induced devaluation effects for foods associated with response inhibition relative to foods that were presented on go trials. In the original experiment we found that devaluation in terms of *explicit* liking was greater for no-go foods than foods that did not appear during training. Preregistered analyses showed that this difference was replicated in the subsequent experiment, but the effect was considerably smaller and only when the data were corrected for outliers the effect size was similar to that reported in Experiment 1 (see **S6.2**). Overall, the study provided evidence for small-to-medium explicit devaluation effects of response inhibition training for appetitive food stimuli and the results presented here indirectly replicated previous findings from studies employing this GNG training paradigm (e.g. see effects reported for a series of experiments in Chen et al., 2016; Chen, Veling, Dijksterhuis, et al., 2018).

### Did the affective priming paradigm capture an ***implicit*** devaluation effect?

Yes. The evaluative categorisation task variant of the affective priming paradigm, which has been previously employed as an indirect measure of food liking (e.g. see Tzavella et al., 2020), was presented after training to assess whether it can capture an *implicit* devaluation effect of response inhibition training. This was achieved by comparing the difference scores from RTs on congruent and incongruent trials (RT priming effect) across training conditions. In both experiments we found that the RT priming effects for no-go food primes were lower compared to go food primes (small-to-medium effects). There was, however, a noteworthy discrepancy between the two experiments for untrained food primes. The RT priming effect for no-go items was reduced relative to the priming effect for untrained items in Experiment 1, but there was only anecdotal evidence for the absence of this expected difference in Experiment 2. Similarly, there was inconclusive evidence for the lack of a positive RT priming effect for untrained items in Experiment 2.

Other studies have shown that ratings for both go and untrained items can decrease and this observation is often attributed to regression to the mean as stimuli with the highest ratings are included in training (e.g. see Chen et al., 2016; Chen, Veling, de Vries, et al., 2018; Quandt et al., 2019). The primary reason for incorporating the second baseline of untrained items in the study design is that effects can become inflated when evaluations for go items are more positive after training (‘go valuation effect’; e.g. see Chen et al., 2016; Chen, Veling, de Vries, et al., 2018). An increase in the evaluations of go items is not very likely given the distributions of ratings for go foods after training and the obtained evidence for an explicit devaluation effect compared to both go and untrained foods. However, we cannot exclude the possibility that the evaluations of untrained items, as measured in the APP, were slightly reduced after training in Experiment 2. The set of stimuli available for selection at the beginning of the study consisted only of energy-dense foods and there were no distinct categories in the training that could explain a transfer of devaluation to untrained items (e.g. low-calorie vs high-calorie) but this issue remains to be formally investigated and results are mixed (see Veling et al., 2017). It is therefore unclear whether certain food items that cannot be classified into different categories but rather different taste profiles (e.g. different flavours of crisps, various types of chocolate bars) would contribute to potential confounds and allow for generalisation of devaluation effects to occur at an ‘implicit’ level. Descriptively we did observe that the change in explicit liking and the RT priming effect for untrained foods were both in the lower range for Experiment 2 relative to Experiment 1. We believe that the generalisation of devaluation effects should be explored more in future experiments as the robustness of this baseline can potentially affect the interpretation of findings in training studies.

There are certain limitations and considerations for future research with regards to the use of implicit measures in response inhibition training studies (also see Tzavella & Chambers, 2022). First, there is not yet enough evidence regarding the sensitivity of the APP in capturing differences in the strength of the evaluations, as for example moderately and strongly liked foods (e.g. see Lamote et al. 2004) or the different components that contribute to ‘implicit’ food attitudes, such as perceived healthiness (e.g. see Tzavella et al. 2020). The sensitivity of implicit measures should be investigated further to appropriately implement them in studies that investigate the mechanisms of action behind training-induced devaluation effects. There may also be methodological challenges in adding implicit measures before and/or after training as response tendencies towards trained items may be affected due to learned associations and it is not yet clear how the strength and duration of the observed devaluation effects are influenced by the order and number of measures presented after training (e.g. see Liu et al. 2022). An outstanding issue in this line of research is also the predictive validity of food evaluation measures and identifying how differently operationalised devaluation effects can predict outcomes in clinical and community-sample studies. We still require more evidence regarding the utility of explicit and implicit measures in predicting real-world eating behaviours (e.g., see Ecological Monetary Assessment study by Masterton et al. 2022).

### What do priming effects imply for theoretical accounts of devaluation effects?

The RT priming effects were inspected on an exploratory basis in Experiment 1 to test whether there was evidence for the expected negative differences between the speed of responses on congruent and incongruent trials. All food items (i.e. primes) were selected to have the highest liking ratings pre-training and this would mean participants should be faster to correctly categorise positive words (congruent) rather than negative words (incongruent). *Post hoc* analyses in Experiment 1 supported our predictions of positive RT priming effects for go and untrained items, but also revealed an interesting trend in the RTs for no-go items, as the priming effect was diminished. In Experiment 2 we addressed these results as part of our preregistered hypotheses and successfully replicated the close-to-zero RT priming effect for no-go foods. This is an important finding of the present research as it has implications for the theoretical accounts of devaluation effects in response inhibition training studies.

Chen et al. (2016) have correctly identified the methodological challenge of measuring ‘implicit’ food evaluations using reaction time tasks, such as the APP, as previous studies have shown that response speed towards no-go stimuli can be reduced after training via learned stimulus-stop associations (Best et al., 2016, 2019; Bowditch et al., 2016). With the present task design, any general slowing of RTs would presumably affect performance in both congruent and incongruent trials and evidence for the absence of a no-go RT priming effect points to the contrary. This could imply that the positive affective reactions towards no-go foods were reduced after training (also see exploratory analyses in **S9**) and in turn the degree of response facilitation in congruent trials and/or response interference on incongruent trials was also decreased (Herring et al., 2013; Wentura & Degner, 2010). This finding can therefore support theoretical accounts of devaluation effects which assume that negative affect is attached to no-go stimuli during training either due to formed stimulus-stop associations and hard-wired connections between go/stop systems and Pavlovian appetitive/aversive centres (Verbruggen et al., 2014; also see Guitart-Masip et al., 2012) or as a result of the conflict that needs to be resolved during training to successfully inhibit responses towards appetitive stimuli (Veling et al., 2008; Chen et al., 2016). While explanatory accounts of the no-go devaluation effect may often overlap (also see discussion in Veling et al. 2017) and the present study cannot provide an answer regarding the specific mechanism of action behind the observed outcomes in training studies, we believe that food evaluations in the laboratory should be further investigated using implicit measures such as the APP not only to shed light into existing theoretical frameworks but also to examine whether we can reliably observe the same effects without the potential limitations of self-report in studies of eating behaviour.

## Conclusion

In this study we report evidence that response inhibition training can lead to the devaluation of appetitive stimuli, such as energy-dense foods and building on previous findings by showing that this effect is not only observed when evaluations are assessed via self-report, but also when they are measured using implicit measures. Specifically, an *implicit* devaluation effect was observed for foods associated with response inhibition during go/no-go training relative to foods that appeared on go trials or were not included in training. Further analyses indicate that the priming effect for no-go foods was close to zero, and this could be attributed to training-induced negative affect for these stimuli. However, we should note that the results of this study should still be interpreted with caution as there may have been a generalisation of the devaluation effect for untrained foods and the implicit measure employed here requires further validation in experimental studies. Future research is required to disentangle theoretical explanations of *explicit* and *implicit* devaluation effects and further evaluate their replicability and factors that could affect their robustness (e.g. generalisation to untrained items, analytical decisions) and sensitivity to experimental manipulation (e.g. differences in training task parameters, the order and duration of post-training behavioural tasks).

## Data Accessibility Statement

Data and analysis scripts for both experiments can be found in our project page on the Open Science Framework (https://osf.io/6bsnv/). All raw data have been made publicly available (DOI: 10.17605/OSF.IO/4DQZB).

## Additional File

The additional file for this article can be found as follows:

10.5334/joc.256.s1 Supplementary Information (SI). s1 to s9.

## Footnotes

In the preregistration for Experiment 1, hypotheses were worded differently so that changes would be “lower” for no-go compared to go and untrained foods, but we have reworded this to aid understanding of the devaluation effects – that is, “lower” does not indicate a smaller effect and a devaluation is expected (i.e., negative change) which would be greater for no-go foods compared to both go and untrained foods. This does not affect the preregistered statistical tests which involve a one-sided negative effect (Measure 1 < Measure 2).

For the Shapiro-Wilk tests we preregistered that supplementary analyses would be performed when the *p*-value was lower than 0.005, which would reflect a less conservative threshold for normality violations.

For the direct comparison of effect sizes, Hedge’s correction was applied to H1a and H1b Cohen’s *d_av_* values to obtain the unbiased effect size estimates (Lakens, 2013) as reported by the authors (Cohen’s *d*_unb_).

Preregistered prediction and justification: Based on the results from our exploratory analyses (Experiment 1), the priming effect for no-go foods may be completely eliminated. However, we could also assume that it is reduced to an extent that only very small effects are obtained.

This recruitment option was anticipated but was omitted in the preregistered protocol.

Supplementary analyses indicated that participants were less accurate on congruent compared to incongruent no-go food prime trials. There was no evidence for speed-accuracy trade-offs in any of the food prime trials (see **S7**).
