# Supplementary material for: Explicit and Implicit Devaluation Effects of Food-Specific Response Inhibition Training
Source: J Cogn. 2023 Jan 17;6(1):10. doi: 10.5334/joc.256 (PMC9854316; doi:10.5334/joc.256)
Supplement: Supplementary Information (SI). — s1 to s9. [file joc-6-1-256-s1.pdf]

Supplementary Information (SI)

S1. Recruitment and data exclusions

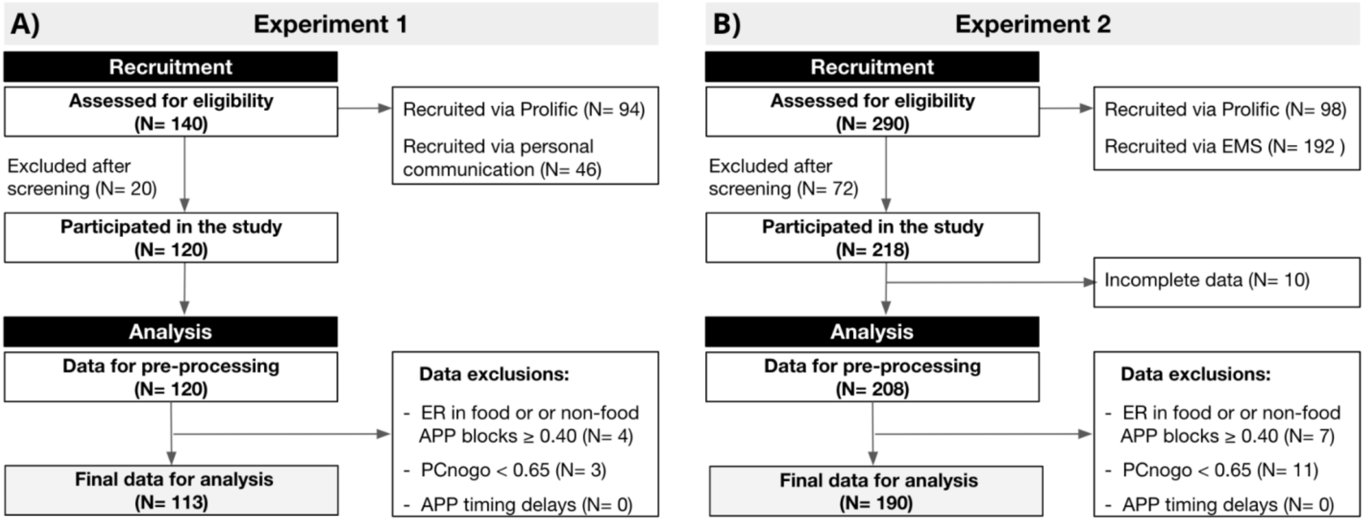

Figure S1. Recruitment and data exclusion diagrams. In the recruitment stage we do not take into account the number of participants who dropped out of the study before it was initiated and only consider the total number of individuals who started the study and answered screening questions to assess their eligibility. In this figure we report the number of participants who had incomplete data (voluntary or software-related drop-outs), were not eligible to participate or were excluded after study completion based on preregistered criteria for data analysis. **A.** In Experiment 1, the majority of participants was recruited via Prolific and a number of individuals from the general population conducted the study online by receiving the link through personal communication. A total of 120 participants were eligible to participate and completed the study. There were minimal exclusions for accuracy in the affective priming paradigm (APP; food or non-food blocks) and the proportion of correct responses on no-go trials in the go/no-go training (GNG) task. After seven exclusions, the final data consisted of 113 participants. **B.** In Experiment 2, the recruitment was extended to include participants from Cardiff University who used the Experimental Management System (EMS) for course credits. A total of 290 participants completed eligibility screening and 72 were excluded although inclusion/exclusion criteria were advertised with the study. From the 218 participants who participated in the study, we received ten datasets that were incomplete (GNG and/or APP data). Out of 208 participants who completed the study, 18 were excluded for their accuracy in the APP and GNG as shown in the diagram. The final data for analysis consisted of 190 participants. There were no data exclusions due to APP timing delays in neither Experiment 1 nor Experiment 2. ER: Error rate(s); PCnogo: Proportion of correct responses on no-go trials

S2. Bayes Factor Design Analyses

For the Bayes Factor Design Analysis (BFDA) we used the BFDA R package (Schönbrodt & Stefan, 2018, 2019) and the code is available at https://osf.io/evcng/. For the BFDA simulations the design priors were the same as the planned analysis priors for both Experiments 1 and 2. For the planned directional Bayesian paired samples-samples t-tests in **Experiment 1** (H1-H3), we examined the probabilities of simulated studies (10,000) terminating at either the boundary for the alternative hypothesis (*H1*) or the null (*H0*) when the specified evidential threshold was reached. Simulations were run for *nmin* (N=50) and *nmax* (N=130) as well as the stopping points for data inspection (i.e., every 20 participants after *nmin* was reached; N=70, N=90 and N=110). These simulations were repeated for three different effect sizes as given by Cohen's *dz* (0.2, 0.5, 0.8) and a true effect of zero (i.e., *H0*).

Analyses for Experiment 1 showed that 62.62% of all simulated correctly terminated at the *H0* boundary when *nmax* was reached and the probability of obtaining false positive evidence was low, with only 0.95% of studies incorrectly terminating at the *H1* boundary. At *nmax*, assuming a 'small' true effect size (*dz*= 0.2), only 35.65% of studies correctly terminated at the *H1* boundary and 4.6% of studies incorrectly terminated at *H1* and no studies reached the *H0* boundary at the specified evidential threshold (0%). For an assumed 'large' true effect size (*dz*= 0.8), 99.99% of studies correctly terminated at *H1* with 70 participants.

The BFDA procedure in **Experiment 2** was adjusted to account for the direct replication of findings and simulations were only run for the expected effect size of 0.35 under *H1* (for justification and details on how this effect size was obtained please see *Analyses* for Experiment 2). The maximum sample size was increased to 200 and consistent with Experiment 1 we used symmetric boundaries for our stopping rule ($BF_{10} \geq 10$ or $BF_{01} \geq 10$). The BFDA was performed for the Bayesian paired samples-samples t-tests that correspond to H1, H2, H3 and H4 in Experiment 2. The R code is available at https://osf.io/esy8x/.

Results showed that 80% of all studies stopped at N= 90 and 95% of all studies stopped at N= 130 if *H1* was true. The false negative rate was 0.6% and 98.4% of studies correctly terminated at the *H1*

boundary. Only 1% of studies terminated at *nmax*, out of which only 0.4% were inconclusive (anecdotal evidence for *H1* or *H0*). Under *H0*, 80% of all studies stopped at N= 130 and 95% of all studies stopped at N= 190. The false positive rate was 2.9% and 88.6% of studies correctly terminated at the *H0* boundary. A total of 8.6% of studies terminated at *nmax* with only 0.4% showing evidence for the alternative hypothesis compared to the null ($BF_{10} > 3$) and 4.7% showing evidence for the null hypothesis compared to the alternative ($BF_{01} > 3$).

S3. Food and non-food stimuli characteristics

The set of food and non-food stimuli used for selection was adopted from previous work that employed the affective priming paradigm as an indirect measure of food liking (see Tzavella et al., 2020 and corresponding Supplementary Material)**.** For food categories that were added in this study we used the food-pics database (Blechert, 2019; Blechert et al., 2014) and Pixabay (https://pixabay.com/). All stimuli that could be shared openly in the public domain are available at https://osf.io/u36nd/ and IDs from the food-pics database are also provided for copyright-protected stimuli (https://osf.io/qbjcg/). The nutritional information of the foods has not been recorded in detail for this study as the task design only included food categories that were considered high in fat, sugar and/or salt according to the NHS colour-labelling system (NHS, 2018) and food choice behaviour was not investigated. Where appropriate, stimuli were edited to have a white background and matched dimensions.

S4. Targets in the affective priming paradigm

At the time of study preregistration, targets were selected based on ratings from 84 individuals recruited as part of ongoing data collection for a previous study (Tzavella et al., 2020). Valence and arousal ratings were recorded, and participants were also asked to indicate which targets they considered ambivalent and if any, such words were excluded (e.g., “alone”). The descriptive statistics for positive and negative target word ratings can be found in Tables S1 and S2 respectively. The sets of positive and negative targets in

our previous study were originally selected from the EMOTE database (Grühn, 2016) and were matched as much as possible on imagery, concreteness, familiarity, and emotionality.

Table S1. Descriptive statistics for *positive* target word valence and arousal ratings

	Valence		Arousal	
Target word	Mean	SD	Mean	SD
BRAVE	7.69	1.15	3.70	1.74
CHEER	7.67	1.16	2.67	1.38
CUTE	7.57	1.58	2.60	1.64
DREAM	7.70	1.12	2.54	1.52
FREE	7.86	1.42	2.36	1.64
FRIEND	8.02	1.20	2.55	1.67
HAPPY	8.16	1.49	2.18	1.59
HOPE	7.87	1.16	2.63	1.35
HUG	7.99	1.15	2.30	1.44
HUMOUR	8.00	1.15	2.18	1.34
INSPIRED	7.82	1.17	2.94	1.60
JOY	7.90	1.26	2.43	1.48
KIND	7.83	1.14	2.36	1.43
KISS	7.72	1.13	2.87	1.62
LAUGHTER	8.01	1.16	2.20	1.35
LOVE	8.42	0.94	2.45	1.88
LOYAL	7.80	1.15	2.73	1.59
LUCKY	7.52	1.29	3.00	1.59
PEACE	8.34	1.05	2.02	1.65
PLEASURE	8.25	1.00	2.25	1.51
PROUD	7.71	1.28	2.93	1.60
SMILE	8.17	1.48	2.36	1.85
SUNSET	7.72	1.17	2.28	1.67
WIN	7.83	1.16	3.33	1.89

Table S2. Descriptive statistics for *negative* target word valence and arousal ratings

	Valence		Arousal	
Target word	Mean	SD	Mean	SD
ABUSE	1.49	0.86	8.11	1.22
AFRAID	2.20	1.37	7.42	1.54
BAD	2.27	1.18	6.86	1.71
BULLY	1.96	1.14	7.40	1.35
CORPSE	1.82	1.12	7.99	1.31
CORRUPT	1.99	1.18	7.30	1.48
CRUEL	1.92	0.99	7.55	1.22
DEATH	1.52	1.05	8.00	1.48
DESPAIR	1.95	1.06	7.63	1.23
EVIL	1.67	1.49	7.83	1.58
FEAR	2.08	1.15	7.66	1.29
GRIEF	1.98	1.31	7.70	1.32
HATE	1.58	1.29	7.67	1.50
HURT	2.12	1.12	7.48	1.36
JAIL	1.92	1.10	7.58	1.53
KILL	1.30	0.87	8.18	1.39
MURDER	1.46	1.24	8.33	1.09
PAIN	2.12	1.31	7.59	1.51
POISON	1.86	1.28	7.66	1.26
SICK	2.11	1.20	7.06	1.65
SLAUGHTER	1.58	0.90	8.00	1.17
TERROR	1.76	1.16	8.11	1.27
TRAUMA	1.82	1.00	7.83	1.23
WAR	1.64	1.08	8.00	1.29

S5. Study questionnaires

Follow-up study questionnaire:

Please answer the following questions about the main word task you completed. Please try to respond honestly. Research shows that people, when answering questions, prefer not to pay attention and minimise their effort as much as possible. If you are reading this, please select "none of the above" on the next question.

Q1. What was this study about?

☐ Food preferences ☐ Attention ☐ Decision-making ☐ None of the above

Q2. Did you notice any differences about your responses on separate occasions? Please select all that apply.

☐ Faster to categorise positive words
☐ Faster to categorise negative words
☐ Faster to categorise words towards the end of the task
☐ Slower to categorise positive words
☐ Slower to categorise negative words
☐ No differences observed

Q3. How frequently did you see the content of the picture that was presented before the word?

1=Never; 2=Very infrequently; 3=Somewhat infrequently; 4=Occasionally; 5=Somewhat frequently; 6=Very frequently; 7=Always

Q4. Please indicate whether you believe that the picture content influenced your responses in any way by selecting all statements below that apply to your performance in the word task.

☐ Faster to categorise positive words when the picture was positive (i.e., picture you liked the most)
☐ Faster to categorise negative words when the picture was negative (i.e., picture you liked the least)
☐ Slower to categorise positive words when the picture was negative
☐ Slower to categorise negative words when the picture was positive
☐ Responses were not influenced by the content of the pictures

Q5. Did you find all the words in the task clearly positive or negative? Certain words may be considered unclear or ambivalent. These may be words that have both positive and negative meaning for you depending on the context. If not, please type in any words in the text box.

- Yes
- No [*open-ended response*]

Q6. Obama was the first American president.

7-point Likert scale (1=strongly disagree to 7=strongly agree)

Q7. Did you purposefully use any kind of strategy to make your responses faster and/or more accurate?

Please select all that apply.

 Slowed down to be more accurate
 Responded fast most of the time and ignored any errors No strategy used
 Other [*open-ended response*]

Q8. How many times were you interrupted during the ATTENTION task (e.g., by the phone ringing or by somebody trying to talk to you)?

 0 1 2 3 4 Other [*numerical input up to two digits*]

Q9. How many times were you interrupted during the WORD task (e.g., by the phone ringing or by somebody trying to talk to you)?

 0 1 2 3 4 Other [*numerical input up to two digits*]

Q10. Were you aware of the study hypotheses/aims prior to completion? If yes, please explain. For example, did you know that the attention task is a form of training and/or what is measured by the word task?

- No
- Yes [open-ended response]

Q11. During the attention task, did you learn that on occasions where you shouldn't respond there were specific food images being shown?

- No
- Yes

All questionnaires added for student analyses were completed at the end of the study in random order and these included the short form of the Barratt Impulsiveness Scale (BIS-15; Spinella, 2007), the Food Cravings Questionnaire - Trait - reduced (FCQ-T-r; Meule et al., 2014), the Perceived Stress Scale (PSS; Cohen et al., 1983), items from the 'Neuroticism' dimension of the Big-Five Inventory (Goldberg, 1993; John & Srivastava, 1999) and the 'weight control', 'health', and 'mood' factors from the Food Choice Questionnaire (Steptoe et al., 1995).

S6. Supplementary analyses for normality violations

S6.1 Normality violations in Experiment 1

Shapiro-Wilk tests revealed that all contrasts (i.e., difference scores of the sample means) under H2 did not follow a normal distribution and as planned, the median RTs were log-transformed and RT priming effects were re-computed for supplementary analyses. Consistent with the results from confirmatory analyses, there was *very strong* evidence for both H2a and H2b. The overall logRT priming effect for no-go foods was lower relative to the observed effect for go foods [H2a; BF_{10} = 67.44; $t(112) = -3.53$, $p < .001$, $d_{av} = -0.37$, 95% CI for $d_{av} = -0.58, -0.16$]. The logRT priming effect for no-go foods was also lower compared to the effect for untrained foods [H2b; BF_{10} = 33.00; $t(112) = -3.30$, $p = 0.001$, d_{av} = −0.31, 95% CI for $d_{av} = -0.50, -0.12$]. The difference scores for changes in liking ratings from pre-to post-training could not be log-transformed, but supplementary analyses were conducted in order to examine whether the devaluation effect was robust after the removal of extreme values in the data. Participants were excluded from these analyses if based on the Interquartile Range Rule (IQR Rule) their difference scores were above or below the acceptable maximum and minimum values[1], respectively. The sample size after the removal of outliers was 103 and there was still *extreme* evidence that participants rated no-go foods more negatively after training relative to both go foods [H1a; BF_{10} = 1126.94; $t(102)$ = −4.37, $p < .001$, $d_{av} = -0.50$, 95% CI for $d_{av} = -0.73, -0.26$] and untrained foods [H1b; BF_{10} = 162.36; $t(102) = -3.81$, $p < .001$, $d_{av} = -0.36$, 95% CI for $d_{av} = -0.56, -0.17$].

S6.2 Normality violations in Experiment 2

RT priming effects under H2 did not violate the normality assumption, as defined in our preregistered exclusion criteria and therefore supplementary analyses were not conducted. On the contrary, Shapiro-Wilk tests suggested deviations from normality for all contrasts under H4. The results from the supplementary analyses with logRTs were consistent with the results from the preregistered analyses. There was *extreme* evidence for a positive RT priming effect when go foods were presented (H4a; BF_{10} =

[1] Any values greater than the third quartile (75%) plus the IQR × 1.5 were removed and any values lower than the first quartile (25%) minus the IQR × 1.5 were also discarded.

661610.51; $t(189) = -5.51$, $p < .001$, $d_{av} = -0.19$, 95% CI for $d_{av} = -0.26, -0.12$), but only *anecdotal* evidence for such an effect when untrained food primes were shown (H4b; $BF_{10} = 0.6972$; $t(189) = -1.80$, $p = 0.037$, $d_{av} = -0.13$, 95% CI for $d_{av} = -0.13, 0.01$). Supplementary analyses indicated stronger evidence for the absence of a positive RT priming effect when no-go food primes were examined (H4c; $BF_{10} = 0.0223$; $t(189) = 0.19$, $p = 0.576$, $d_{av} = 0.01$, 95% CI for $d_{av} = -0.06, 0.07$).

After removal of outliers based on the IQR Rule for changes in explicit liking from pre-to post-training, there were 173 participants in the sample. The supplementary analyses were not consistent with the preregistered analyses without outlier exclusion with regards to untrained foods. There was *strong* evidence that participants rated no-go foods more negatively after training compared to untrained foods [H1b; $BF_{10} = 10.36$; $t(172) = -2.69$, $p = 0.004$, $d_{av} = -0.19$, 95% CI for $d_{av} = -0.34, -0.05$]. After exclusions for outliers, there was still *extreme* evidence that the change in explicit liking for no-go foods was more negative relative to the change for go foods [H1a; $BF_{10} = 888.80$; $t(172) = -3.94$, $p < .001$, $d_{av} = -0.30$, 95% CI for $d_{av} = -0.45, -0.15$].

S7. Inspection for speed-accuracy trade-offs

Error rates on congruent and incongruent trials across training conditions were inspected for the potential occurrence of speed-accuracy trade-offs (SATs), where participants strategically choose between accuracy and speed (e.g., slowing down to be more accurate, or responding consistently fast and ignoring any errors). Error rates would need to be either greater on incongruent compared to congruent trials or statistically no difference should be observed. Bayesian and frequentist paired samples t-tests were not directional (or two-tailed). We used the same analyses priors employed for preregistered analyses in the two experiments.

In Experiment 1, there was *moderate* evidence that error rates did not differ between congruent ($M = 0.07$, $SD = 0.07$) and incongruent go food prime trials ($M = 0.08$, $SD = 0.07$) [$BF_{01} = 4.75$; $t(112) = -1.20$, $p = 0.232$]. On no-go food prime trials, there was *moderate* evidence for no differences in error rates between congruent ($M = 0.07$, $SD = 0.07$) and incongruent trials ($M = 0.07$, $SD = 0.07$) [$BF_{01} = 6.65$;

$t(112) = 0.87$, $p = 0.388$]. *Moderate* evidence for the null compared to the alternative was also obtained for error rate differences on congruent ($M = 0.07$, $SD = 0.07$) and incongruent untrained food prime trials ($M = 0.07$, $SD = 0.07$) [$BF_{01} = 6.85$; $t(112) = -0.83$, $p = 0.408$].

In Experiment 2, there was again *moderate* evidence that there was no difference between error rates on congruent ($M = 0.07$, $SD = 0.06$) and incongruent go food prime trials ($M = 0.08$, $SD = 0.07$) [$BF_{01} = 9.92$; $t(189) = -0.88$, $p = 0.381$]. There was only *anecdotal* evidence that error rates did not differ between congruent ($M = 0.09$, $SD = 0.07$) and incongruent no-go food prime trials ($M = 0.07$, $SD = 0.07$) [$BF_{01} = 2.37$; $t(189) = 2.94$, $p = 0.004$]. A follow-up test was performed and showed that there was *strong* evidence that on average participants had more errors on congruent compared to incongruent no-go food prime trials [$BF_{10} = 20.12$, $p = 0.002$]. For untrained food prime trials, there was *strong* evidence for no difference between error rates on congruent ($M = 0.08$, $SD = 0.07$) and incongruent trials ($M = 0.08$, $SD = 0.07$) [$BF_{01} = 20.70$; $t(189) = -0.34$, $p = 0.735$].

S8. Robustness checks for analysis prior in Experiment 1

To assess how robust our results were to the choice of a default Cauchy prior in the first Experiment we first conducted checks using JASP to examine whether evidence for our hypotheses – as indicated by the computed BFs - was changed when a wide and ultrawide prior was used (see Figure S2). None of the directional t-tests had any meaningful differences in terms of the strength of evidence for the alternative compared to the null hypothesis. For our manipulation check (H3) with an ultrawide prior the BF was 93.87 (i.e., just under the threshold for *extreme* evidence). After the checks were completed, we recomputed all BFs for the pre-registered hypotheses using the updated informed prior from Experiment 2 and additional location parameters (see Table S3) to account for the smallest effect size of interest (d=0.163; smaller effect reported in Study 2 of Chen, Veling, Dijksterhuis et al. 2018 for low value food items) and the average expected effect size that has informed the sampling plan in similar studies (d=0.537; e.g., Chen, Veling, de Vries et al. 2018; Chen et al. 2019).

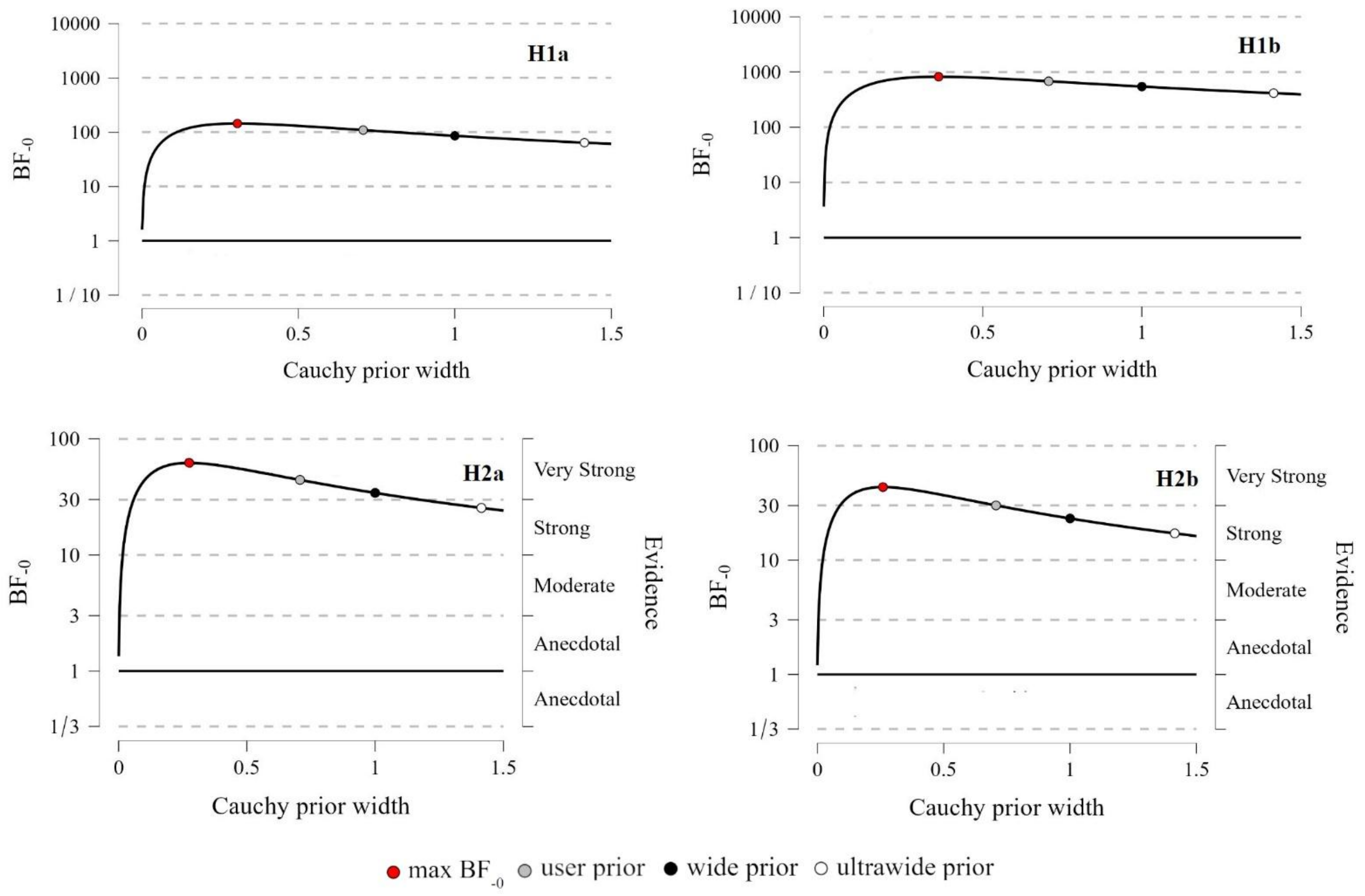

Figure S2. Robustness check plots for Cauchy priors in Experiment 1. The computed Bayes Factors for the directional hypotheses (BF_{-0} for Measure 1 < Measure 2) were not meaningfully affected in terms of strength of evidence for H1a and H1b – other than a marginal reduction from *extreme* to *very strong* evidence for H1a. Although there is still conclusive support for H2a and H2b as shown in the plots the relative strength of evidence for the alternative compared to the null hypothesis is slightly reduced when the width of the Cauchy prior increases (i.e., from our selected default – user prior to wide and ultrawide prior).

Table S3. T-test results for Experiment 1 with different informed priors

Analysis prior	BF_{10}				
	H1a	H1b	H2a	H2b	H3
Original Cauchy prior (Experiment 1)	109.42	678.73	44.30	30.06	158.99
Informed prior (t=-0.163; scale=0.102; df=3)	197.88	845.05	95.44	69.54	266.80
Informed prior (t=-0.350; scale=0.102; df=3)	393.87	2435.62	152.03	99.80	577.67
Informed prior (t=-0.537; scale=0.102; df=3)	171.26	1619.36	53.43	32.04	274.28

S9. Priming effects for low and high liking ratings

To what extent might the washing out of the no-go priming effect reflect the elimination of response tendencies after training rather than attenuated evaluations of the food stimuli? To answer this question, we undertook an exploratory analysis to compare the training-induced reduction of the priming effect between foods that achieved lower vs. higher liking ratings. We reasoned that if training eliminated response tendencies (independently of food devaluation) then the reduction of the no-go priming effect should be insensitive to liking ratings. On the other hand, if training caused devaluation then the reduction should be greater for lower-rated foods (see Figure S3 below). As the experiments did not differ in their task designs or procedure, for this analysis we pooled the data across Experiment 1 and 2 to maximise sensitivity (N=303). In each training condition there were only 8 food items, represented by two exemplars. This meant that there were not enough available food categories to extract a ‘moderate’ and ‘high’ liking spectrum of scores, so instead we applied a median split to assign all food items in each condition into low and high categories. This was undertaken for both pre-training and post-training ratings.

As a first step in this analysis, we examined the distribution of mean liking ratings from the pre-training phase and excluded participants who had a negative average (<0) for no-go or untrained foods as this would indicate an overall disliking of the items (N=243) . Similarly, we excluded extremely positive values (>80) as they could not be considered moderate (N=230). Next, we calculated the average RT

priming effect (ΔRT) for lower-rated and higher-rated foods in the no-go and untrained conditions (see Figure S3) based on the ratings acquired post-training. Our first assumption for this analysis was that no-go foods that were rated more negatively would be easier to devalue and would therefore show a greater devaluation effect compared to lower-rated untrained foods. As shown in Figure S3, we expected that this devaluation effect for lower-rated foods (no-go < untrained) would be greater than that for higher-rated foods. Therefore, our next step in this analysis was to calculate the two priming effects directly (untrained – no-go for lower-rated foods and untrained – no-go for higher-rated foods). If a no-go priming effect was completely diminished due to items not eliciting any response tendencies, we would expect no difference between the two devaluation effects (RT priming differences). However, if the devaluation effects reported as part of our pre-registered analyses were driven by a change in the strength of liking for no-go stimuli then we would expect that the devaluation effect for lower-rated items would be greater than that for higher-rated items. In the final step of the analysis we further removed outliers for the calculated difference scores based on the IQR rule, as previously explained in S6.1, which resulted in a sample of 215 participants.

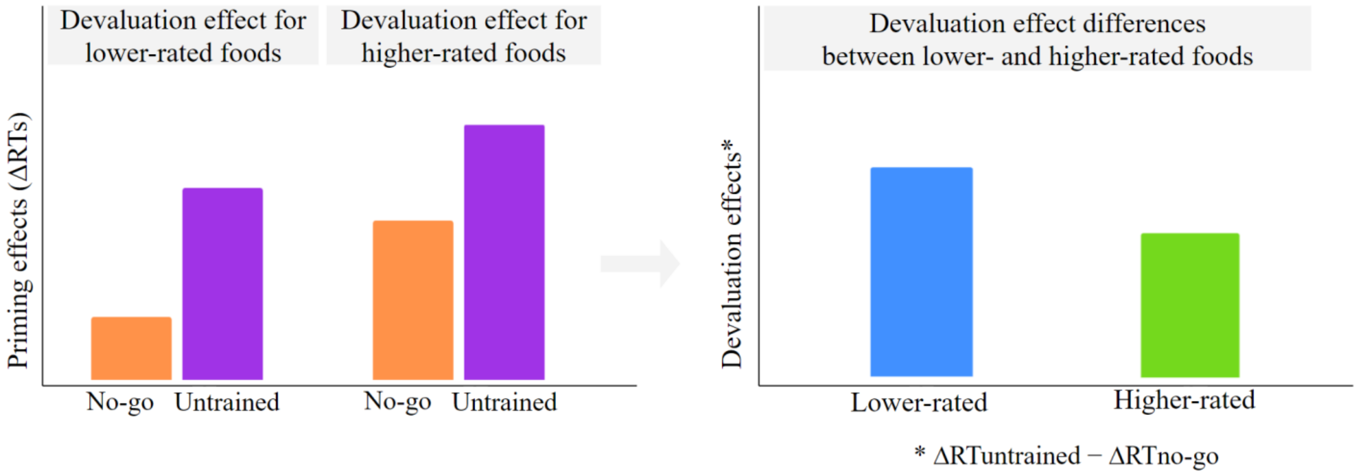

Figure S3. Predicted differences between devaluation effects for lower- and higher-rated foods if the training influenced valuation of the foods rather than a generalised reduction of response tendencies.

We conducted Bayesian paired samples t-tests with the informed prior parameters from Experiment 2 and assuming a small effect (0.20) for the expected difference (devaluation effect for lower-rated foods > devaluation effect for higher-rated foods). Supplementary frequentist statistics are provided for

completeness. Consistent with the devaluation hypothesis, there was *moderate* evidence that the priming devaluation effect for lower-rated foods post-training (*M* = 15.67, *SE* = 3.29) was greater than that for higher-rated foods (*M* = 5.28, *SE* = 3.47) [BF_{10} = 8.30; *t*(214) = 2.37, *p* = 0.009, *d* = 0.16; also see Figure S4]. Looking at each devaluation effect separately, there was *extreme* evidence for a small-to-medium effect for lower-rated items as the ΔRT was smaller for no-go (*M* = -7.45, *SD* = 45.04) compared to untrained foods (*M* = 8.23, *SD* = 41.04) [BF_{10} = 16755.15; *t*(214) = -4.77, *p* = < .001, *d* = -0.33]. For higher-rated items, there was only *anecdotal* evidence for a very small effect and the ΔRT was slightly smaller for no-go (*M* = 4.74, *SD* = 46.95) compared to untrained foods (*M* = 10.02, *SD* = 46.19) [BF_{10} = 1.25; *t*(214) = -1.52, *p* = 0.064, *d* = -0.10]. Overall, this analysis suggests that training influenced the strength of liking for no-go stimuli rather than causing a non-specific elimination of response tendencies.

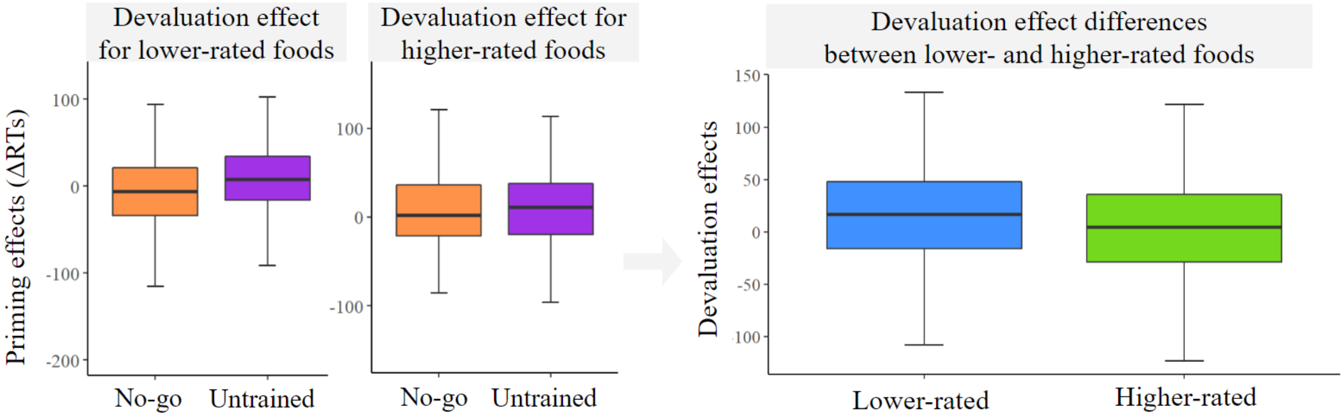

Figure S4. Plotted devaluation effects for lower- and higher-rated foods. Vertical bars in the boxplots indicate the range, excluding the outliers based on the Interquartile Range.

SI References

Blechert, J. (2019). Food-Pics_Extended—An Image Database for Experimental Research on Eating and Appetite: Additional Images, Normative Ratings and an Updated Review. *Frontiers in Psychology*, *10*(307), 1–9.

Blechert, J., Meule, A., Busch, N. A., & Ohla, K. (2014). Food-pics: An image database for experimental research on eating and appetite. *Frontiers in Psychology*, *5*(617), 1–10. https://doi.org/10.3389/fpsyg.2014.00617

Chen, Z., Veling, H., de Vries, S. P., Bijvank, B. O., Janssen, I. M. C., Dijksterhuis, A., & Holland, R. W. (2018). Go/no-go training changes food evaluation in both morbidly obese and normal-weight individuals. *Journal of Consulting and Clinical Psychology*, *86*(12), 980–990.

https://doi.org/10.1037/ccp0000320

Chen, Z., Veling, H., Dijksterhuis, A., & Holland, R. W. (2018). Do impulsive individuals benefit more from food Go/No-Go training? Testing the role of inhibition capacity in the no-go devaluation effect. *Appetite*, *124*, 99–110. https://doi.org/10.1016/j.appet.2017.04.024

Chen, Z., Holland, R. W., Quandt, J., Dijksterhuis, A., & Veling, H. (2019). When mere action versus inaction leads to robust preference change. *Journal of Personality and Social Psychology*. https://doi.org/10.1037/pspa0000158

Cohen, S., Kamarck, T., & Mermelstein, R. (1983). A global measure of perceived stress. *Journal of Health and Social Behavior*, *24*(4), 385–396.

Goldberg, L. R. (1993). The structure of phenotypic personality traits. *American Psychologist*, *48*(1), 26–34. https://doi.org/10.1037/0003-066X.48.1.26

Grühn, D. (2016). An English Word Database of EMOtional TErms (EMOTE). *Psychological Reports*, *119*(1), 290–308. https://doi.org/10.1177/0033294116658474

John, O. P., & Srivastava, S. (1999). The Big Five Trait taxonomy: History, measurement, and theoretical perspectives. In *Handbook of personality: Theory and research, 2nd ed* (pp. 102–138). Guilford Press.

Meule, A., Hermann, T., & Kübler, A. (2014). A short version of the food cravings questionnaire-trait: The FCQ-T-Reduced. *Frontiers in Psychology*, *5*(190), 1–10. https://doi.org/10.3389/fpsyg.2014.00190

NHS. (2018, April 27). *Food labels*. https://www.nhs.uk/live-well/eat-well/how-to-read-food-labels/

Schönbrodt, F. D., & Stefan, A. M. (2018). *BFDA: An R package for Bayes factor design analysis (version 0.4.0) [Computer software]*.

Schönbrodt, F. D., & Stefan, A. M. (2019). *BFDA: An R package for Bayes factor design analysis* (0.5.0) [Computer software]. https://github.com/nicebread/BFDA

Spinella, M. (2007). Normative Data and a Short Form of the Barratt Impulsiveness Scale. *International Journal of Neuroscience*, *117*(3), 359–368. https://doi.org/10.1080/00207450600588881

Steptoe, A., Pollard, T. M., & Wardle, J. (1995). Development of a Measure of the Motives Underlying the Selection of Food: The Food Choice Questionnaire. *Appetite*, *25*(3), 267–284. https://doi.org/10.1006/appe.1995.0061

Tzavella, L., Maizey, L., Lawrence, A. D., & Chambers, C. D. (2020). The affective priming paradigm as an indirect measure of food attitudes and related choice behaviour. *Psychonomic Bulletin & Review*, *27*(6), 1397–1415. https://doi.org/10.3758/s13423-020-01764-1
